# A Comparative Analysis of Data Synthesis Techniques to Improve Classification Accuracy of Raman Spectroscopy Data

**DOI:** 10.1021/acs.jcim.3c00761

**Published:** 2023-10-11

**Authors:** Aaron R. Flanagan, Frank G. Glavin

**Affiliations:** School of Computer Science, University of Galway, Co. Galway H91 FYH2, Ireland

## Abstract

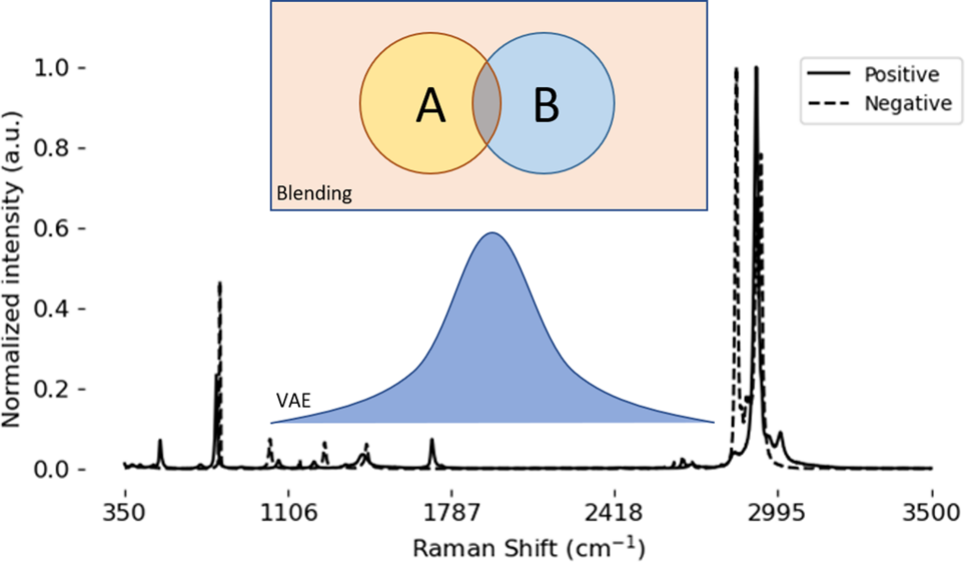

Raman spectra are examples of high dimensional data that can often be limited in the number of samples. This is a primary concern when Deep Learning frameworks are developed for tasks such as chemical species identification, quantification, and diagnostics. Open-source data are difficult to obtain and often sparse; furthermore, the collecting and curating of new spectra require expertise and resources. Deep generative modeling utilizes Deep Learning architectures to approximate high dimensional distributions and aims to generate realistic synthetic data. The evaluation of the data and the performance of the deep models is usually conducted on a per-task basis and provides no indication of an increase to robustness, or generalization, on a wider scale. In this study, we compare the benefits and limitations of a standard statistical approach to data synthesis (*weighted blending*) with a popular deep generative model, the *Variational Autoencoder*. Two binary data sets are divided into 3-fold to simulate small, limited samples. Synthetic data distributions are created per fold using the two methods and then augmented into the training of two Deep Learning algorithms, a *Convolutional Neural Network* and a *Fully-Connected Neural Network*. The goal of this study is to observe the trends in learning as synthetic data are continually augmented to the training data in increasing batches. To determine the impact of each synthetic method, *Principal Component Analysis* and the *discrete Fréchet distance* are implemented to visualize and measure the distance between the source and synthetic distributions along with the Machine Learning metric *balanced accuracy* for evaluating performance on imbalanced data.

## 1 Introduction

Raman spectroscopy is a key instrument for collecting and analyzing detailed information about chemical composition and structure. A monochromatic laser illuminates a sample and measures the difference in the frequency between the incident beam and scattered light. Raman spectra represent the intensity of the scattered light as a function of this frequency shift, which is referred to as the *Raman shift*.^1^ In the excitation phase, elastic scattering is observed when the molecules in the sample absorb the radiation and shift to a virtual energy state before relaxing back to their ground state and emitting the radiation. Raman spectroscopy is the observed effect of inelastic scattering; this is when the intensity of the incident light becomes lower or higher after the molecules relax. During this phase, the molecules exhibit unique vibrational modes that are dependent on the incident wavelength.^2^ The unique interactions between light and matter enable Raman spectroscopy to be an effective method for the qualitative and quantitative analysis of chemical species. The spectral patterns recorded from this process function as a *fingerprint* that can be used for identification of the sample. Furthermore, the intensity of the radiation can be measured to quantify the concentration or presence of a target analyte in the compound.

A large and extensive collection of literature has investigated the pairing of Raman spectroscopy with Machine Learning to automate the analysis phase. Some examples include evaluating *k-nearest neighbors (kNN)*, *Neural Networks (NN)*, and *Partial Least Squares (PLS)* as suitable methods for predicting the concentration of narcotics, comparing traditional chemometric analysis techniques and Machine Learning algorithms in classifying chlorinated compounds, and the identification of cell death with *Support Vector Machines (SVM)*.^3−5^ The issue with traditional chemometrics and Machine Learning is that preprocessing for cosmic ray removal, smoothing, baseline correction, and dimensionality reduction is typically required to achieve reliable performance. Recently, studies have been focused on the application of Deep Learning algorithms because they can perform automatic feature selection and do not require this preprocessing step. Deep Learning algorithms mitigate problems such as manual feature engineering; however, issues such as the availability of data and resources block advancements in these applications too. Data that are publicly available can often be sparse or limited, and if new data are required, they can be costly to collect and curate, requiring a high level of expertise and time. Practitioners should always challenge the veracity of the data too. A common mistake is the introduction of bias by nonexperts in the training phase. Spectral data can also contain hundreds and thousands of dimensions but are limited in the number of samples available. Deep Learning algorithms do not generalize well in this scenario due to a phenomena referred to as the *curse of dimensionality*.^6^ This observation states that to approximate some function the number of samples required grows exponentially as the dimensions increase.

Data synthesis acts as a supplemental method for data acquisition and offers an effective way of enhancing the spectral database and augmenting the training data used for Deep Learning algorithms. Synthetic data are capable of modeling realistic samples that include variation not observed during the initial collection process while increasing the number of samples that can be used for comparison. If we consider that each real-world sample *N* takes *T* amount of time to observe, collect, and categorize, then expansion of the spectral database naturally, while maintaining the same level of quality, is *T* * *N*. When applying data synthesis techniques, the majority of the effort is invested in the design and experimentation phases; however, once finalized, a computational model can generate synthetic spectra in constant time. This reduces the cost, time, and expertise required to curate the data and build intelligent solutions.

In this work, we compare two data synthesis techniques and their performance on two Deep Learning models under the assumption of limited data. The data synthesis methods include a statistical approach referred to as *weighted blending* and a deep generative modeling technique, the *Variational Autoencoder (VAE)*.^7^ We train a *Convolutional Neural Network (CNN)* and a *Fully-Connected Neural Network (FCNN)* on two Raman spectra data sets for the task of identifying *chlorinated vs nonchlorinated solvents* and the task of identifying *healthy vs positive patients* with the *SARS-CoV-2 virus*.

## 2 Literature Review

A large and continuously growing body of literature has investigated ways of using Raman spectra to solve complex tasks and, in tandem, improvements to Deep Learning methods for optimizing the efficiency and performance on such tasks. Conroy et al.^4^ present work in comparing traditional chemometric analysis techniques and Machine Learning to Raman spectra. The aim was to determine the suitability of Raman spectroscopy to correctly classify chlorinated and nonchlorinated solvents. The chemometric analysis techniques included *Principal Component Analysis (PCA)* and *Principal Component Regression (PCR)*, with methods such as max normalization, and first and second derivative preprocessing. This was compared to four Machine Learning algorithms which included *kNN*, *SVM*, *RIPPER*, and *C4.5 Decision Tree*. It was reported that the traditional approach achieved an error rate of 5%, which was achieved with second derivative preprocessing applied to a restricted spectral range. The Machine Learning algorithms outperformed the traditional approach, with the SVM algorithm achieving an error rate of 4.8% without any hyperparameter tuning. The final observation was that the Machine Learning algorithms performed similarly and, in certain situations, slightly better than the traditional techniques with room for more experimentation and improvement. This data set was also previously used in studies by Howley et al.,^8^ Glavin and Madden,^9^ and Khan and Madden^10^ for investigating methods such as PCA, one-class classification, and custom similarity functions for improving the classification performance of high dimensional data in Machine Learning algorithms.

A recent study proposes serum-based Raman spectra for rapid and accurate screening of patients infected with the *SARS-CoV-2 virus*.^11^ Traditional methods such as real-time polymerase chain reaction (RT-PCR) tests are time-consuming and susceptible to high false negatives. To overcome this constraint, the researchers propose using a serum extracted from the blood samples of confirmed infected, suspected, and healthy patients and converting this via Raman spectroscopy for identification and classification. An *Analysis of variance (ANOVA)* feature selection algorithm is applied to select the relevant features that improve the classification performance of an SVM. The experiments are designed to compare each class (healthy; positive; suspected) in a binary fashion. The authors report that, on average, the healthy versus positive classification achieves 91% accuracy. The positive versus suspected classification achieves a smaller 86% accuracy, while the suspected versus healthy classification is only 69%. Zhao et al.^12^ combined a population-based optimization technique called Grey wolf optimization (GWO) with an SVM, reporting a test accuracy of 90.8%. Krohling and Krohling^13^ propose the combination of a Savitzky–Golay filter for spectral preprocessing and a 1D-CNN for classification, reporting an average accuracy of 96%.

Liu et al.^14^ evaluated the first application of a Deep Learning classification approach that can manage raw spectra, which typically required a considerable amount of preprocessing. A CNN was compared to standard Machine Learning methods for the classification of minerals. Each algorithm was trained on the processed spectra of 1671 minerals from the RRUFF^15^ database. The CNN is reported to outperform each method in the top-1, 3, and 5 accuracy scores, achieving a top-1 accuracy of 88% with the SVM performing second at 81%. In the second set of experiments, six baseline correction methods were applied to a set of 512 unprocessed raw spectra. The authors report that the CNN not only outperforms the other methods but also a decrease in performance is observed when baseline correction is applied. This was further demonstrated by applying the same method to another independent mineral data set and observing the same results.

Fan et al.^16^ proposed a Deep Learning-based component identification framework (DeepCID) for the task of single component identification in mixtures via raw Raman spectra. DeepCID is a collection of one-class classification models each trained to discriminate among 1 of 167 pure compounds and filter out baseline, fluorescence, and noise. This framework was compared to five other standard classification algorithms including a kNN, *Random Forest (RF)*, *Logistic Regression (LR)*, *Back propagation Artificial Neural Network (BP-ANN)*, and a FCNN. DeepCID is reported to perform better than the other algorithms, with the LR model performing comparably, but also includes achievements such as zero false positives and negatives for powder, liquid, and ternary mixtures. Furthermore, 7 models achieved an accuracy of no less than 98.8%, with 160 achieving over 99.5%.

Ho et al.^17^ apply Deep Learning for the task of identifying bacterial species as a solution to reduce broad spectrum antibiotic treatments. A CNN is trained on 60,000 spectra, comprising 30 bacterial and yeast isolates modeled from 94% of the treated infections in Stanford Hospital between 2016 and 2017, and 12,000 isolates from clinical patients. The isolate-level accuracy is reported at 82.2%, compared to LR at 75.7% and an SVM at 74.9%. The authors also conclude that by grouping known antibiotic treatments and bacterial species, the average classification accuracy can be extended to 97%, compared to LR at 93.3% and SVM at 92.2%.

A smaller segment of the literature concentrates on deep generative modeling as a supplemental method for performing qualitative analysis by filtering, encoding, and synthesizing new data. In the work preceding this study, Houston et al.^18^ compared weighted blending (see section 3.5.1) and a VAE (see section 3.5.2) for increasing classification performance and model robustness on the Raman spectra of chlorinated compounds. This included techniques such as one-class classification, binary classification, and a two-step classification approach for outlier detection. A kNN, SVM, and a custom designed *Locally Connected Neural Network (LCNN)* were used to evaluate performance on real, synthetic, and real plus synthetic data for the weighted blending and VAE. It is reported that the kNN accuracy decreased in all scenarios, from 91.3% on the real data to 90.7% for both the blended and blended plus real data, and 89% and 90.1% for the VAE only and VAE plus real data. The SVM performance on real data was 93%, dropping to 92.4% and increasing up to 94.6% for the blended and blended plus real data. The accuracy increased to 93.6% and 94.2% for the VAE only and VAE plus real data. The deep LCNN model received the largest benefit increasing from the baseline 96.1% to 97.6% and 97.3% for the blended and blended plus real data set, with the lowest result being 92.6% for the VAE only data set and the highest recorded accuracy of 98% for the real plus VAE synthetic data. The authors hypothesize that the addition of synthetic spectra reduces the model’s tendency to overfit, resulting in more confident predictions.

In a similar study, Liu et al.^19^ combined a VAE and *Long Short-Term Memory (LSTM)*(20) network for bacterial pathogen classification. Five bacterial pathogens are used to train the VAE for synthetic data generation. The source and synthetic data are combined and used to train the LSTM model to perform classification. The VAE encodes and maps the source data points to a Gaussian distribution and reconstructs the spectra, which results in decreasing noise and increasing the signal-to-noise ratio (SNR). This has the effect of increasing the classification performance in the LSTM network by reducing noise and outliers. The authors report that the VAE-LSTM combination has a mean accuracy of 96.9% at classifying over 16 pathogenic bacteria at the strain level across 5 distinct species.

He at al.^21^ proposed a similar framework by training a VAE to compress, clean, and reproduce the spectra of cancer subtypes at the cellular and tissue level before performing classification. The VAE automatically filters randomly occurring noise, disregards redundant features, ignores outliers, and manages the high dimensionality of spectral data without a decrease in performance. The results of nine Machine Learning algorithms are reported, showing an improvement when trained on the VAE synthetic data. The top two models included *Gaussian Naive Bayes (NB)* increasing from 81% to 89% and a linear SVM increasing from 83.3% to 88.6% for cellular level classification. The authors also report an increase from 77.5% to 81.4% for Gaussian NB on kidney cancer spectra for tissue level classification.

While not related to the methods applied in this study, other approaches are being investigated for data synthesis and Deep Learning that demonstrate promising results. For example, Shang et al.^22^ demonstrated that Deep Learning can be applied in conjunction with multiple detection technologies. Using fluorescence imaging and Raman spectroscopy, data were collected from the breast tissue of 14 patients, with each sample generating 120 fluorescence images. Pseudocolor enhancement was applied, and a pretrained GoogLeNet^23^ CNN was tasked to classify if the sample is cancerous or healthy, obtaining a reported accuracy of 88.61%. Two FCNNs were trained on the Raman spectra, obtaining 95.33% and 98.67%. The predictions of the fluorescence images and Raman spectra were combined into a characteristic variable matrix and further predicted using a *Partial Least Squares (PLS)* algorithm. The authors conclude that merging the predictive results, selecting the appropriate threshold value for comparison, and training the PLS model obtain an accuracy of 100%.

Yu et al.^24^ developed an LSTM network for the rapid identification of marine pathogens via Raman spectra. Eight strains were collected from the Urechis unicinctus larvae marine organism, covering four pathogens, and used to train an LSTM and CNN. It is reported that the LSTM achieves a mean strain level accuracy of 94%, compared to the CNN’s 89%. The authors concluded that the LSTM has a reduced training time and increases accuracy over that of the state-of-the-art. A custom designed loss function is also presented to improve the explainability of the features in the Raman spectra that contribute to the classification. By constructing a phylogenetic tree, the genetic distance between strains was obtained and inserted into the model as the training label, thus enforcing the model to learn the genetic distribution values between strains.

Teng et al.^25^ developed a *Generative Adversarial Network (GAN)* to generate synthetic data for laser-induced breakdown spectroscopy (LIBS). To evaluate the quality of the synthetic spectra, two unsupervised clustering techniques, *K-Means* and PCA, were applied. The quality is measured as the percentage of synthetic spectra clustered with real data, ranging between 59–100% for five samples, with two samples achieving 100% overlap of their respective source. The authors note that the GAN is not capable of representing physical meaning, such as the theoretical bounds of fluctuations in the spectral lines or noise introduced from the environment; it also requires up to 180,000 training iterations to approximate the distribution and generate similar looking spectra.

In a follow-up study, Yu et al.^26^ developed a GAN to generate synthetic data for improving the classification performance on marine pathogens. Individual GANs were developed and trained on 50 random samples per strain as the target and 100 samples of the two remaining strains labeled as fake. The authors report that the models converge around 30,000 epochs of training; this is when the generator model starts to produce synthetic data that are similar to the source strain. It is also reported that the discriminator maintains a 100% classification accuracy between the strains when evaluated on separate test data, indicating that this approach can balance the training between the two models and maintain a high-level of performance for generation and discrimination.

Wu et al.^27^ propose a framework for using a GAN and CNN for skin cancer vs normal tissue classification. The authors state that training Deep Learning models is a challenge and propose a pipeline infrastructure to address the issue. A data set was curated containing three categories with limited samples, including basil cell carcinoma (36), squamous cell carcinoma (50), and normal cells (60). The experiments are conducted using a stepwise augmentation on two forms of synthetic training sets: a balanced data set with an equal number of samples per category and a stratified set where the class prior probability is maintained. A mix of 12 Machine Learning and Deep Learning models is trained and compared to the framework, with a subset of these methods also having data augmentation applied. The authors report that the deep models outperform the standard in all scenarios, that the augmented methods outperform their respective baselines, and that their approach outperforms all other methods in most cases.

## 3 Materials and Methods

### 3.1 Sample Preparation

The first data set used in this study contains 230 Raman spectra originally curated by Conroy et al.^4^ and enhanced by Howley et al.^8^ for the quantitative and qualitative analysis of chlorinated solvents with Machine Learning. The spectra were obtained from the mixtures of 25 chlorinated and nonchlorinated solvents of various grades mixed in different concentrations as outlined in Table 1. As reported by the authors, the spectral measurements were captured via a Labram Infinity (J-Y Horiba) spectrometer equipped with a liquid nitrogen cooled CCD detector and a 488 nm Argon ion laser, with an average power output of 7 mW. Each spectrum is an average recorded over 3 × 10 s exposures in the 350–3500 cm^–1^ range. After collection, the spectra are smoothed using the Savitzky–Golay derivative preprocessing technique. This method performs a 7-point averaging least-squares fit of the data using a second degree polynomial to smooth the spectra for baseline correction.

**Table 1:** Summary of the Grades Used for Chlorinated and Nonchlorinated Solvents^18^

Solvents	Chlorinated	Nonchlorinated	Total
Pure	6	24	30
Binary Mix	96	23	119
Ternary Mix	40	12	52
Quaternary Mix	12	10	22
Quintary Mix	0	7	7
No. of Spectra	**154**	**76**	**230**

The second data set was provided by Yin et al.^11^ from their work studying the efficiency of Raman spectroscopy and Machine Learning to screen positive cases of the SARS-CoV-2 virus. Raman spectra were collected from confirmed healthy and negative patients at Sichuan Cancer Hospital and Chengdu Public Health Clinical Medical Center. The data contain 150 spectra from healthy patients and 159 spectra from patients confirmed carrying the SARS-CoV-2 virus. The authors report that the spectra were measured using a volume-phase holographic (VPH) spectrograph with a deep-cooled CCD camera, Raman probe, and single-mode diode laser set at 785 nm. The spectra are measured over 15 × 3 s exposures per sample at 70 mW, ranging between 600–1800 cm^–1^. Polynomial fitting and normalization by total area were employed for baseline correction, and the automatic-weighted least-squares algorithm was applied for spectral smoothing.

### 3.2 Data Preprocessing

Baseline correction and spectral smoothing algorithms are important preprocessing steps for removing or reducing interference effects and noise in the true Raman spectrum. Raman spectroscopy can be influenced by various factors that have the potential to alter the spectral signal and impact multivariate analysis and calibration models. These include sample-related causes such as fluorescence or the presence of impurities in the target, instrumentation-related problems such as electrical noise leading to low-frequency fluctuations in the spectral signal, or external factors such as cosmic radiation or excessive light interfering with the spectrometer’s detector.^28,29^ Popular algorithms for smoothing and baseline removal include iterative least-squares algorithms, polynomial fitting, wavelet transforms, and moving averages such as the Savitzky-Golay filter.^30^

**Figure 1:**
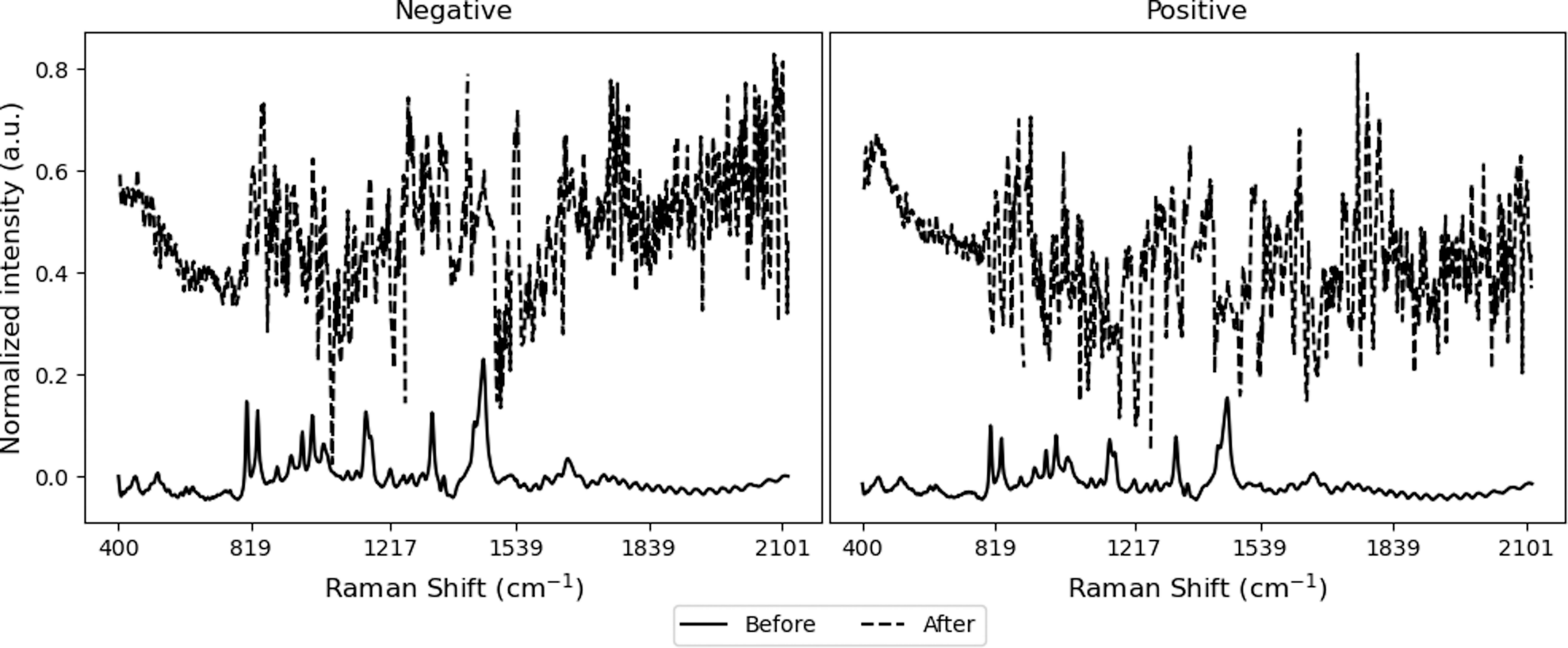
Samples of positive and negative SARS-CoV-2 Raman spectra before and after min-max normalization.

A side effect of the spectral smoothing and baseline correction of the SARS-CoV-2 data resulted in a subset of the features containing only zero intensities. These features would not contribute to the results reported by the authors due to the Analysis of Variance (ANOVA) method applied for feature selection in their work. However, feature selection is not required for Deep Learning, as it is inherent, and the features present an issue for further rescaling of the data. The SARS-CoV-2 data contain negative values, and we require them to be in a positive range for two reasons: (1) for fair comparison of classification performance on multiple data sets, the data formats should be as similar as possible, (2) Deep Learning algorithms may formulate patterns or correlations to the zero valued features that do not exist. To achieve these requirements, we remove eight feature columns and apply a min-max normalization to shift the range of the data from −0.1–0.4 to between 0–1, as shown in Figure 1 which illustrates the spectral structure of a negative and positive SARS-CoV-2 sample pre- and postprocessing.

**Figure 2:**
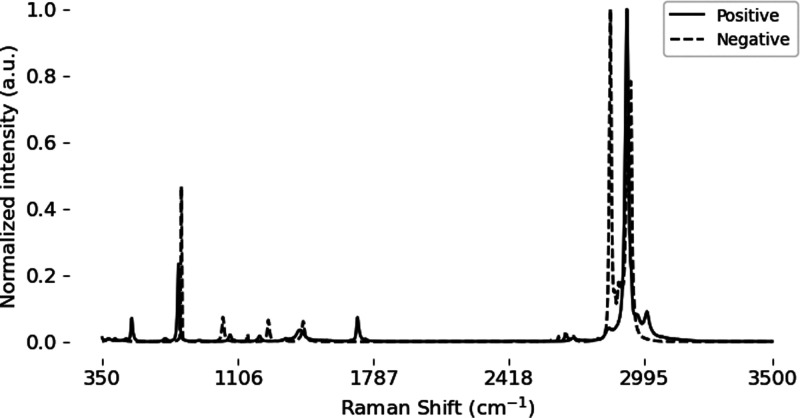
Example of positive and negative Raman spectra of chlorinated compounds after min-max normalization.

The chlorinated data are already min-max normalized between 0–1 with 2473 dimensions, as shown in Figure 2; no further preprocessing was applied.

### 3.3 Cross-Validation

**Figure 3:**
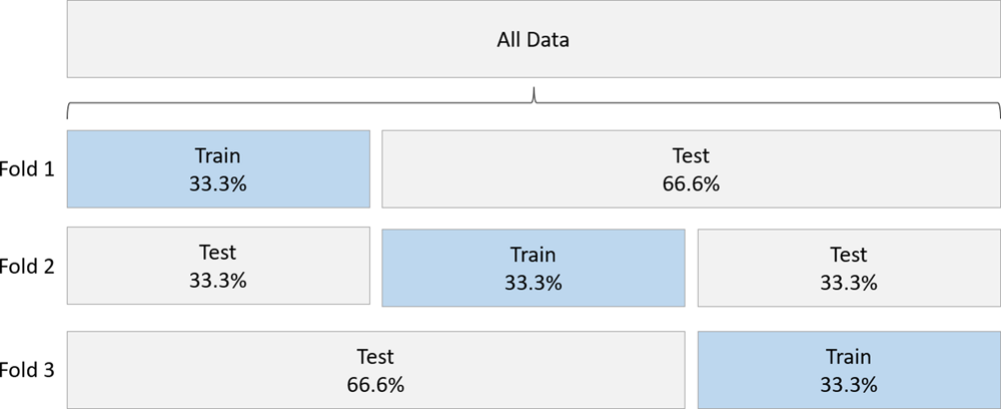
Division of training and testing into three stratified nonoverlapping folds.

It is crucial to evaluate Deep Learning models using representative real-world data. Synthetic data are generated under assumptions, may lack representation, and may not fully capture the inherent nuances or idiosyncrasies of real-world data such as noise and outliers. Deep Learning algorithms tend to overfit and memorize their training data, so the evaluation of model performance with synthetic data will set unrealistic expectations. For this reason, and due to the higher number of training samples made available via the synthetic methods, we opted to store a larger proportion of the principal data as a holdout set so that algorithm performance will be assessed using a realistic proportion of only *real-world* data. Furthermore, evaluating and averaging the results of each algorithm on various unique data sets provide a strong indication of the algorithm’s robustness and generalization to the task. So, we also apply a 3-fold cross-validation by dividing each principal data set into three stratified and nonoverlapping folds, as shown in Figure 3. The principal data are divided such that each fold contains 33.3% of the unique real spectra used for training and synthetic data generation. The remaining 66.6% of the real spectra are used as a holdout set for evaluation. This process was conducted once before experimentation, with the unique folds and corresponding holdout sets stored to disk. The exact spectra selected for each fold are stored and published in the data folder of the repository linked in the Data and Software Availability section below.

For the SARS-CoV-2 data set, each training fold contains 103 samples in total, with a 51% positive (53 samples) and 49% negative (50 samples) class prior probability. Each respective holdout set contains 206 samples with the class prior probability maintained. The chlorinated data set contains 230 samples, which results in an extra two samples, one positive and one negative, in the final fold. To evenly divide the chlorinated data, one random sample is dropped for each class, and the remaining 228 spectra are split with each training fold containing a 67% positive (51 samples) and 33% negative (25 samples) class prior probability. Each respective holdout set contains 154 samples with the class prior probability maintained.

### 3.4 Generating Synthetic Data

**Figure 4:**
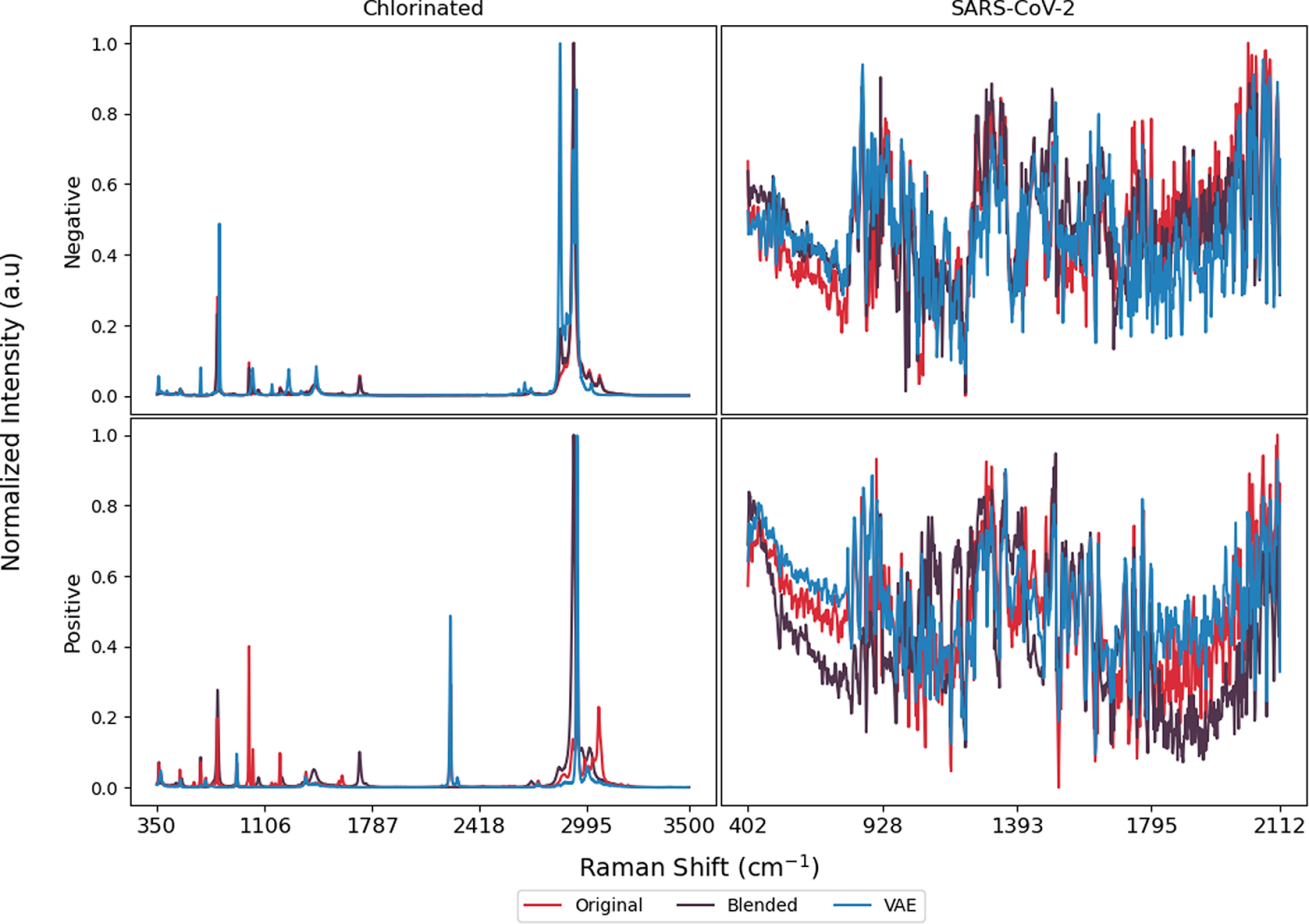
Random samples from the first training fold including original spectra after preprocessing and generated synthetic data.

The synthetic data distributions are created on a per-fold basis with each fold producing two unique synthetic data sets (see sections 3.5.1 and 3.5.2) generated from the 33.3% training data of that fold. In the experiments, a new model is initialized per-fold so that it will only have visibility of 33.3% of the data during training. This includes when the training fold is augmented with synthetic data because the data are sampled from the respective synthetic distributions created using the 33.3% training data of that fold. Figure 4 illustrates randomly sampled spectra from the first training fold and the respective synthetic data sets.

### 3.5 Data Synthesis

#### 3.5.1 Weighted Blending

Weighted blending is a technique demonstrated by Houston et al.^18^ as an efficient method for synthetic data generation and improving classification performance of Deep Learning models. Blending multiple spectra results in new synthetic data that can be used for modeling variation, database enhancement, and training of Deep Learning models.

##### Implementation

Weighting is implemented by taking the product of each intensity value and a set of static weights θ independently over *N* parent spectra. Synthetic spectra are then produced by calculating the sum of the weighted intensities of the parent spectra at the corresponding wavelengths. In this work, we apply blending of two parent spectra *A* and *B* using nine weights. If *S*_*n*_ is the set of unique synthetic spectra, and θ is a set of weights ranging from 0.1 to 0.9 (inclusive), *S*_*n*_ can be generated as shown in eq 1.
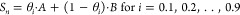
1

The formula represents an iterative dot product and summation of weights in θ applied to the intensity values at corresponding wavelengths across parent spectra *A* and *B*. The number of unique spectra produced per class is determined by the number of weights and can be calculated using the formula |θ|*N*(*N* – 1)/2, where |θ| is the length of θ, and *N* is the number of samples. The number of unique spectra generated is 14175 and 23427 for the chlorinated and SARS-CoV-2 data, respectively.

#### 3.5.2 Variational Autoencoder

Variational Autoencoders (VAEs) are probabilistic generative Deep Learning models that perform variational inference to approximate a sample distribution and generate new synthetic samples.^7^ This is achieved using two neural networks termed the *encoder* and *decoder*. The encoder *E* creates latent representations of the input attributes by learning a mapping from the input space, *p*(*x*), to a latent space distribution, *q*(*z*), parametrized by two encoding vectors μ̂ and σ̂. The generation of synthetic data is performed by the decoder *D* which samples from these vectors, adds noise ϵ, and up-samples the values to produce a new output, *x̂*. The goal of the network is to maximize information retention when encoding and minimize the reconstruction loss when decoding.

##### Implementation

**Figure 5:**
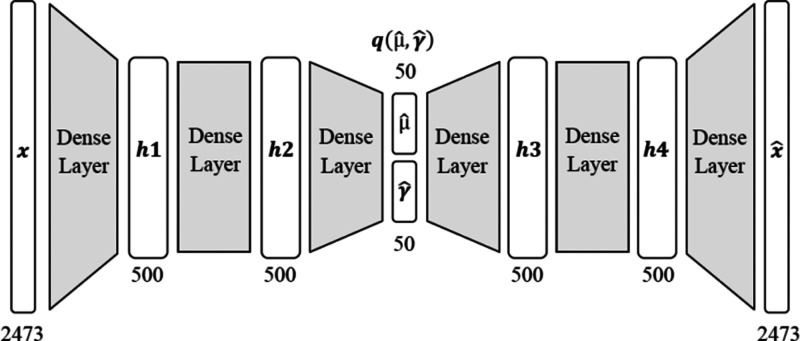
Architecture of the Variational Autoencoder as illustrated by Houston et al.^18^

The implementation of the VAE, as shown in Figure 5, is based partially on Geron (2017).^31^ Models *E* and *D* contain two hidden layers with 500 neurons using an exponential linear unit (ELU) activation function. The latent space encoding vectors μ̂ and γ̂ is represented as two hidden layers with 50 neurons and no activation, and γ is the log of the encoding vector σ. The noise ϵ is sampled from a normal distribution with μ = 0 and σ = 1 and applied to the sample vector in the following format (eq 2):
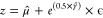
2

Both models are trained for 10,000 epochs using the Adam^32^ optimizer with a learning rate of 0.001 and default beta parameters. The reconstruction loss is binary cross-entropy, and the Kullback–Leibler (KL) divergence^33^ is added as a latent loss to penalize the model if it diverges from the desired latent space. The cost function (eq 3) is defined as the reconstruction loss plus latent loss:
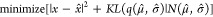
3

### 3.6 Deep Learning

#### 3.6.1 Convolutional Neural Network

Convolutional Neural Networks (CNNs) are a state-of-the-art Deep Learning technique inspired by the biological visual cortex.^34^ CNNs are a class of feed-forward Artificial Neural Networks (ANNs) that include convolution and pooling layers for down-sampling the input and learning low-level abstract features such as edges and lines. This is achieved via *n* × *m* matrices, referred to as kernels, which are convolved across the input and down-sampled in the pooling layers to remove redundant information. The abstract features enable the network to learn high-level patterns in deeper layers that are forward-propagated through fully connected layers to make a final prediction. Furthermore, convolution layers include parameter sharing via the kernels, thus reducing the number of parameters required during training and introducing locality to the learning process. This provides several advantages over standard feed-forward models: (1) CNNs become scale and translation invariant, (2) they are robust in large dimensions, and (3) including local context makes CNNs highly suitable to pattern and image recognition tasks.

##### Implementation

Our implementation is based on the CNN design in the DeepCID framework proposed by Fan et al.^16^ for single component identification in mixtures. The model architecture consists of two down-sampling blocks each containing convolution, max-pooling, and dropout layers, respectively, followed by two fully connected layers and the output layer. The first fully connected layer size is determined by the number of output values from the final dropout layer ×64 feature maps. The second fully connected layer had 1024 hidden units. The weights of the network are initialized as a truncated normal distribution with μ = 0 and σ = 0.1; the bias is a constant value set to 0.1. The Rectified Linear Unit (ReLU) activation function is used in all layers, and the output activation function is changed from a softmax to sigmoid to adapt the multiclass design to binary for this work.

#### 3.6.2 Fully-Connected Neural Network

Fully-Connected Neural Networks (FCNNs), otherwise known as Artificial Neural Networks (ANNs), are a family of Deep Learning models in which layers of neurons are stacked sequentially with each neuron connecting to every neuron in the subsequent layer. Each neuron performs a weighted sum of inputs that is wrapped in an activation function, to introduce nonlinear interpolation, before forward-propagating to the next layer. This allows such models to learn nonlinear relationships between the input features and is referred to as the Universal Approximation Theorem.^35−37^ This theorem states that an FCNN with a single hidden layer and an infinite number of neurons can approximate any continuous function that is bounded to a specific range and nonconstant.

##### Implementation

**Figure 6:**
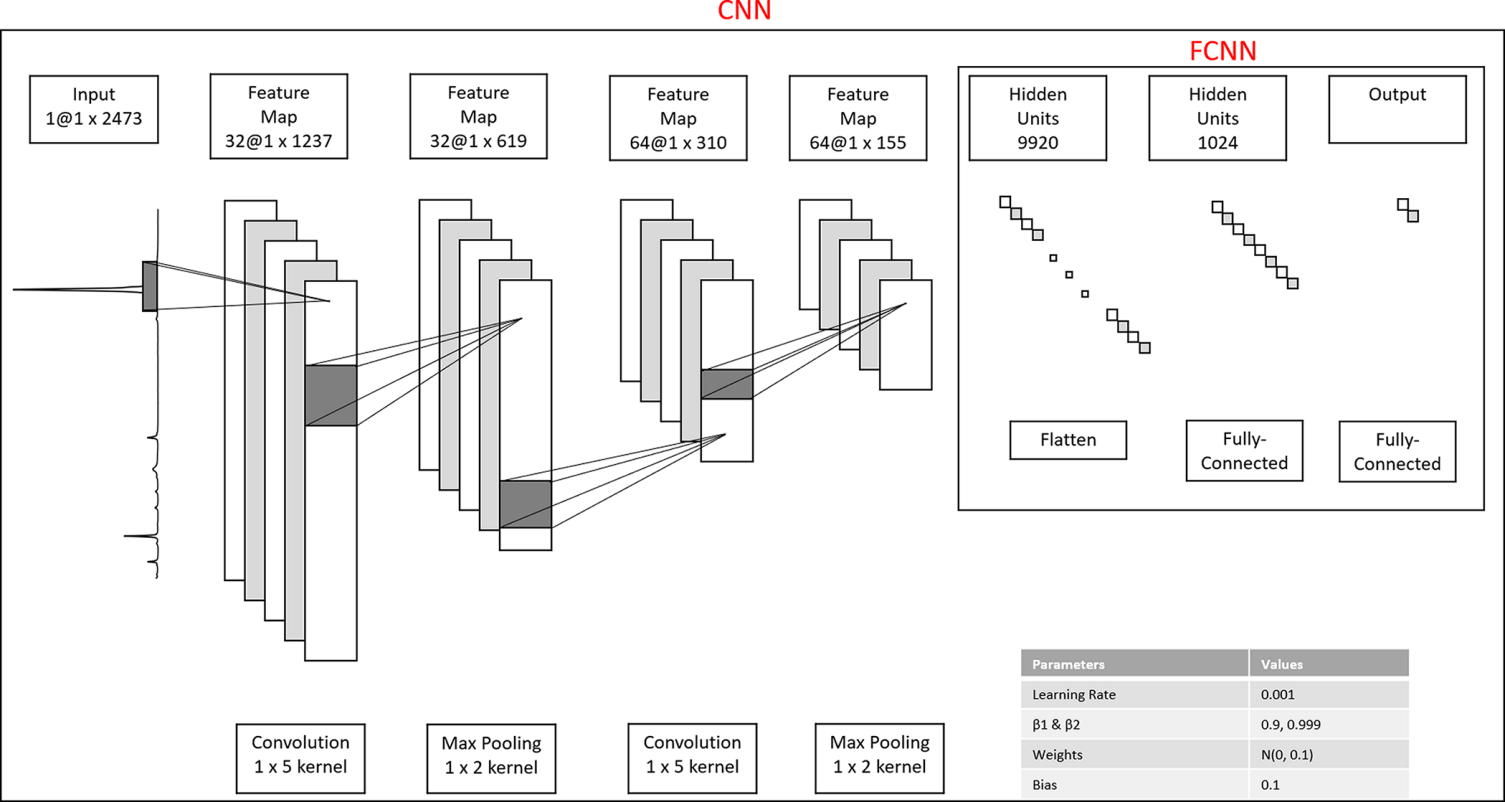
Architecture of the CNN applied to a chlorinated sample. The FCNN is obtained by removing the two convolutional blocks.^16^

The FCNN model is created by removing the convolutional blocks of the CNN previously described, leaving only two fully connected layers and the output layer. The first hidden layer size is equal to the input dimensions of the sample data, and the second layer has 1024 hidden units. The weights, bias, optimizer, learning rate, and activation functions are the same as those of the CNN, as illustrated in Figure 6.

## 4 Results and Discussion

To perform comparative analysis, the algorithms are first trained on the principal data sets to record a baseline. For each synthetic technique, training is performed on both algorithms in a stepwise fashion by augmenting the data with synthetic spectra. The training set is increased by 400 samples per step with the maximum number of samples set at 2000. In each step, both algorithms are trained on the 3-fold for five runs each using seed values 0–4. For each seed, a model is created and trained using the current training fold and evaluated with the respective fold’s holdout set. The models are trained for 100 epochs using the Adam^32^ optimizer with default hyperparameters and a learning rate of 0.0001. The cost function is binary cross entropy with a batch size of 1. The results disclosed are averaged over the five seeds (runs) per fold, which is further averaged over the three folds.

For a fair comparison, the algorithms will not be tuned during experimentation, and each model will be trained as described above. The goal of this study is to observe the learning trends when synthetic data are introduced. To achieve state-of-the-art performances, hyperparameter tuning and model architecture adjustment would be required to find the best hypothesis that can approximate the distributions. This study focuses on the impact on performance with respect to the synthetic data. The following sections describe how the synthetic methods and algorithms are evaluated along with the results and corresponding discussions for each approach.

### 4.1 Evaluation of Synthetic Spectra

Each synthetic data generation method has several advantages and disadvantages that must be considered before implementing them in a productive capacity. These depend on factors such as data availability, the proportion of data, budget, equipment, business needs, and research purposes. Deep generative modeling is a time-consuming endeavor, where the best results that can be obtained are a product of sourcing relevant literature and extensive testing. We compare the synthetic methods by drawing observations from their respective synthetic distributions and provide a discussion regarding their influence on the two deep learning algorithms.

The quality of the synthetic spectra is evaluated before experimentation using a distance-based metric and PCA. We first measure the distance between the real and synthetic distributions, per fold, by applying the *discrete Fréchet distance* (dFid).^38^ The dFid, or coupling distance, approximates the *Fréchet distance* for determining the similarity between curves. Given two curves, *P* and *Q*, all pairs of points *pi* and *qi* are observed, and the Euclidean distance is calculated for each pair. Restrictions are applied to reduce the computational complexity by allowing only three paths: both *pi* and *qi* take one step forward, *pi* remains static and *qi* moves forward, or *pi* moves forward and *qi* remains static. The result is the maximum of all of the minimum distances required to traverse both curves. The dFid path walking algorithm is applied to the mean spectra calculated from the training and synthetic data per fold. The results are presented below in Table 3 and Table 5 in the discussion section of each method.

**Figure 7:**
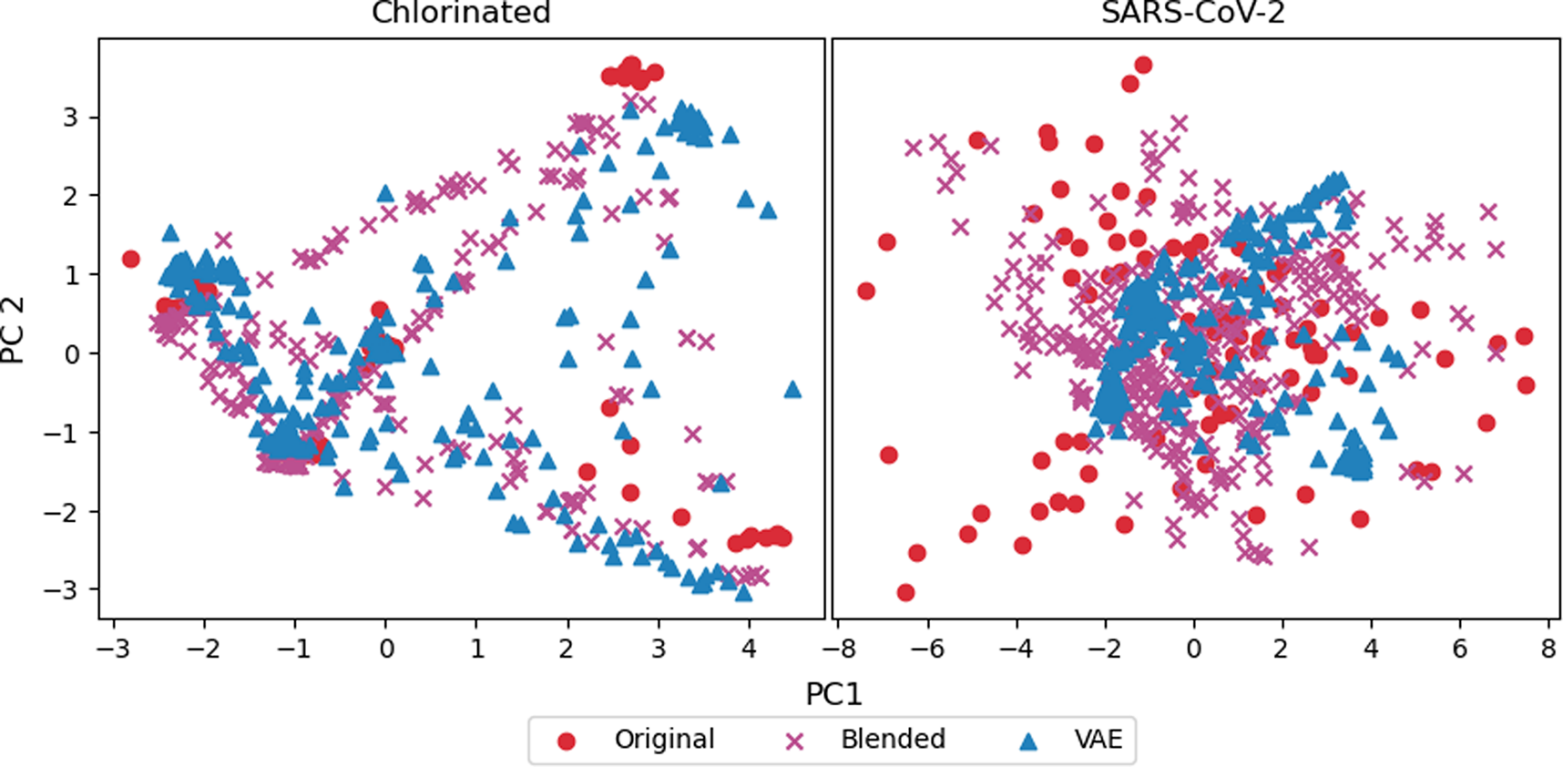
PCA plot of original and synthetic samples from the first training fold.

The second quality test is a visual comparison of the spectra after projecting the data points to a lower 2-dimensional space using PCA, as shown in Figure 7. This plot contains the PCA scores for two principal components calculated from the first training fold and the respective synthetic data sets. The synthetic spectra are randomly sampled at three times the size of the original spectra to avoid oversaturation due to the high number of synthetic spectra available. The objective of PCA is to maximize the variation by projecting the data onto new axes in an orthogonal fashion. For the chlorinated data on the left, the projected points for the original data are clustered in four distinct locations, with the blending and VAE PCA scores following a broadcasting motion between the points. This indicates that the methods are likely influenced by distinct features that dominate the feature space of the data, such as peak location and magnitude, or dense areas such as the chlorinated region of the spectra between 350–1168 cm^–1^.^4^ On the right side, the PCA scores for the original SARS-CoV-2 data are spread out over a larger range with less structure. The blending PCA scores support this having a similar distribution; however, the VAE PCA scores are more centered, indicating that the model is capable of identifying outliers or noise and reducing them when reconstructing the samples.

### 4.2 Evaluation of Deep Learning Models

As previously mentioned, the chlorinated data contain an imbalance with 67% of the data containing positive instances. Sensitivity and specificity are two metrics for measuring the percentage of correctly identified samples conditioned on all of the true samples in the data. Sensitivity measures the percentage of correctly classified positive samples, as shown in eq 4, and is often referred to as the True Positive Rate (TPR).
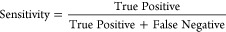
4Specificity measures the percentage of correctly classified negative instances, as shown in eq 5, and is termed the True Negative Rate (TNR).
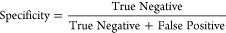
5Balanced accuracy is then defined as the arithmetic mean of these metrics, as shown in eq 6, providing a balanced interpretation of a model’s ability to discriminate between the class instances. It can also be described as the average total recall for all classes present in the data.
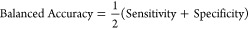
6

### 4.3 Baseline

Referring to Table 2, it can be observed that the algorithms underperformed on the chlorinated data. The CNN model had an average test accuracy of 56.1%, which is only slightly better than random. When setting the model to evaluation mode, the training set predictions result in an accuracy score of 73.8%, 72.1%, and 79.7% for each fold, respectively. The model failed to learn the data, and as a result, performance was poor on the holdout sets. On the other hand, the FCNN achieved 98.2% for fold one and 100% accuracy for folds two and three on the training data, obtaining a final mean test accuracy of 77.5%. While this indicates the FCNN models improved the ability to discriminate between the classes, the error rate is still large at 22.5%. The chlorinated data are so large and complex that neither model is able to approximate its distribution with small, limited sample sets.

**Table 2:** Balanced Accuracy Test Results Obtained on Principal Data

Data	Model	Balanced Accuracy
Chlorinated	CNN	0.561 ± 0.041
	FCNN	0.775 ± 0.025
SARS-CoV-2	CNN	0.864 ± 0.020
	FCNN	0.849 ± 0.029

In comparison, the SARS-CoV-2 models produced much better results, but it should be noted that these data contain more samples and have 1500+ fewer dimensions. These data are also more balanced with a class prior probability of 51% and 49% for positive and negative (healthy), respectively. When evaluating the CNN and FCNN on the training samples for each fold, the model converged to 100% for the first two folds, and for the final fold, the CNN achieved 96.7%, and the FCNN achieved 99.4%. The final average test accuracy for the models is much better than the chlorinated data at 86.4% and 84.9%, respectively. Given that the data are more balanced and contain more samples, and the dimensionality is one-third of the chlorinated data, it is not surprising that the models did not overfit to the same degree. However, an error rate between 13.6 and 15.1% is still very large given the severity of the consequences for an incorrect classification, regardless of whether the patient is positive or negative. It is apparent from the baseline results that the data sets contain too few samples for the algorithms to learn from, and as a result, they do not generalize well.

### 4.4 Blending

The blending method is a simple and practical way of generating synthetic data for experimentation, and due to the simplistic implementation of the technique, the synthetic distribution will be a close approximation of the source, with the advantage that any set of arbitrary weights can be applied to control the amount of variation. This can be observed in Table 3 outlining the rounded dFid scores for both data sets. It should be noted that the dFid scores are range- and scale-dependent. In this context, the chlorinated data resulted in a larger dFid because the feature values were initially spread over a larger range before normalization was applied. It does not indicate that the SARS-CoV-2 data were a better fit because both data sets are produced under the same weighting scheme.

**Table 3:** dFid Scores of Blended Synthetic Spectra

Fold	Chlorinated	SARS
1	0.034	0.005
2	0.034	0.005
3	0.030	0.004
Average	**0.033**	**0.005**

Blending is deterministic and also relatively quick when compared to deep generative models. Applying a specific weighting scheme guarantees to produce the same distribution each time, which takes, on average, 12–15 min per data set with nine weights and 100 samples each. The reproducibility and fast production time make it a suitable method for spectral database enhancement. However, this approach has limitations; as the number of weights and resulting synthetic spectra increases, it requires larger amounts of unique and real examples to model appropriate synthetic data. It will also require manually constructed restrictions to generate spectra that model the real population of samples, such as upper and lower thresholds for fluctuations in intensities at peaks. Another side effect to consider is if a particular class of spectra contains more examples, with larger weight sets, the resulting synthetic spectra will become oversaturated with the dominant class. When data are generated using blending, an even number of samples per class should be maintained.

From the results outlined in Table 2 and Table 4, we observe inverse effects on the models. The chlorinated CNN improved from the average test accuracy of 56.1% (baseline, with no synthetic example additions) to 77.4% after 400 samples are augmented, and the best result achieved at 1200 synthetic samples was 79.4%. However, the standard deviation over the 15 (5 seeds × 3 folds) iterations per augmentation step ranged between 2.3 and 5.5%, containing a lot of variation in between folds and between the iterations per fold. Evaluating the model on the training samples shows an average of 99% accuracy up to 1200 samples and 100% thereafter with smaller deviations in all scenarios. The model is capable of learning and overfitting on the data with only a few hundred extra samples added. It is evident the CNN suffered from the curse of dimensionality in the baseline and that augmenting the chlorinated data with synthetic samples had a positive effect, but it still does not generalize well with an error rate of 20.6–22.6%. The FCNN followed a similar pattern, overfitting and converging to 100% accuracy when evaluated on the training samples in all cases but only receiving a marginal increase in performance on the test cases. The deviations are slightly smaller, indicating an increase in robustness, but this can be considered negligible when the baseline 77.5% accuracy with a 2.5% deviation is compared to the augmentation results. The average test accuracies after augmentation range between 77.7 and 78.5% with a 21.5 and 22.3% error rate. Considering the added cost of synthesizing new samples and the increased training time of the algorithms, the blending method can be considered to have had no impact on the performance of chlorinated FCNN model’s performance.

**Table 4:** Test Balanced Accuracy Scores from Augmenting Blended Synthetic Spectra

		Chlorinated	SARS
Model	Samples	Balanced Accuracy	
CNN	400	0.774 ± 0.023	0.842 ± 0.023
	800	0.783 ± 0.050	0.853 ± 0.016
	1200	0.794 ± 0.045	0.851 ± 0.014
	1600	0.788 ± 0.045	0.851 ± 0.015
	2000	0.779 ± 0.055	0.85 ± 0.019
FCNN	400	0.777 ± 0.016	0.859 ± 0.011
	800	0.785 ± 0.010	0.845 ± 0.037
	1200	0.781 ± 0.011	0.861 ± 0.011
	1600	0.779 ± 0.011	0.861 ± 0.012
	2000	0.779 ± 0.011	0.859 ± 0.014

In contrast to the chlorinated CNN, the SARS-CoV-2 CNN received no positive increase in performance, with the baseline accuracy dropping from 86.4% with a 2% deviation to the best achieved of 85.3% with a 1.6% deviation at 800 samples. The training evaluation was 100% in all cases; however, the baseline produced similar results, resulting in the model receiving no improvements after augmentation. The FCNN did receive improved performance, increasing from 84.9% with a 2.9% deviation between folds to 86.1% with a 1.1% deviation at 1600 samples. We hypothesize that the blended data did not include enough variation to increase the robustness of the models to the unseen samples, and as a result, the performance of the CNN converged around 1200 samples for the chlorinated data and negatively impacted the SARS-CoV-2 data. The FCNN models both had a marginal increase in performance; however, for the chlorinated data, the accuracy range remained the same. A small improvement was seen in its robustness due to the deviations between the folds shrinking. From these results, we can conclude that blending is a quick and useful method to extend the spectral database, and it has the potential to increase the classification ability of deep models; but the improvement is limited by the number of unique samples available and the complexity of the task. In certain cases, such as SARS-CoV-2, the performance of more complex models like a CNN might be impacted due to the low representational power of blended synthetic data generated from such limited sample sets.

### 4.5 VAE

One of the main advantages of the VAE is the restriction imposed by the latent space. The low dimensionality restricts the VAE from modeling random noise and outliers. The spectra are modeled as a distribution, and the KL regularization term added to the loss function penalizes the model if the distributions diverge. This characteristic restricts the model so that it only retains relevant information and discards the noise.^21^ The VAE thus acts as a filter learning to compress the input, remove noise, and ignore outliers. When reconstructing a sample, the random noise ϵ that is added in the sampling step introduces variation to create new synthetic spectra. Another benefit is that deep generative models can overcome class imbalances because the practitioner has the ability to specify the number of synthetic spectra generated per class, allowing balanced data sets to be created before training the deep algorithms.

When comparing it to the blending technique, the VAE requires more training time, is nondeterministic, and requires an exhaustive search of the parameter space. However, by altering the model architecture, latent space design, and the amount of noise added, the VAE can be designed to produce fluctuations in the spectra that keep the new samples in a realistic sample space. Even with limited training data, the VAE can produce realistic synthetic spectra in larger amounts and with a more controlled variation. This can be observed in Table 5 where the average dFids are 0.044 and 0.029. Deep generative methods such as the VAE are inherently stochastic; so to measure the general distance between the distributions, we generated new synthetic distributions and averaged the dFid scores over five iterations per fold.

**Table 5:** dFid Scores of VAE Synthetic Data

Fold	Chlorinated	SARS-CoV-2
1	0.031	0.026
2	0.035	0.029
3	0.066	0.033
Average	**0.044**	**0.029**

Augmentation of the VAE synthetic data shows similar results, presented in Table 6, for the CNN models with a positive trend for the chlorinated data and negative impact for SARS-CoV-2. Similar to the blended approach, the model training scores for the chlorinated data range between 99 and 100%, with the lowest being 95%; however, with the VAE spectra, the chlorinated CNN has continuous improvements from the baseline 56.1% up to 82.2% at 2000 samples. The chlorinated FCNN also has improved results using the VAE spectra, showing continuous increases from 1200 to 2000 samples, moving from 80.3 to 81% at 2000 samples. The decrease in the deviations between training folds from 2.5% in the baseline to a maximum of 2.2% at 2000 samples indicates a minor increase in robustness too. The VAE synthetic chlorinated spectra had a more influential effect on the results for both models.

**Table 6:** Test Balanced Accuracy Scores from Augmenting VAE Synthetic Data

		Chlorinated	SARS
Model	Samples	Balanced Accuracy	
CNN	400	0.769 ± 0.043	0.842 ± 0.026
	800	0.793 ± 0.034	0.831 ± 0.020
	1200	0.800 ± 0.039	0.829 ± 0.015
	1600	0.804 ± 0.055	0.830 ± 0.019
	2000	0.822 ± 0.018	0.824 ± 0.027
FCNN	400	0.801 ± 0.017	0.849 ± 0.016
	800	0.798 ± 0.015	0.851 ± 0.011
	1200	0.803 ± 0.021	0.848 ± 0.010
	1600	0.807 ± 0.017	0.854 ± 0.006
	2000	0.810 ± 0.022	0.846 ± 0.013

The SARS-CoV-2 CNN training scores converged to 100% accuracy with 0% variance in all cases, but the test scores indicate a reduction in the models’ robustness, continually decreasing from the baseline 86.2% to a low 82.4% at 2000 samples. Referring to Figure 7, it can be observed in the PCA graph that the VAE synthetic data lack variation when compared to the original data. The VAE may have failed to capture the true representation of the principal data which as a consequence impacted the models’ learning. The FCNN results varied between 84–85%, neither better nor worse than the baselines 84.9%. The CNN and FCNN did not benefit from the VAE SARS synthetic spectra, likely due to the lack of new samples and variation.

## 5 Conclusion

The properties and information obtained from Raman spectroscopy make it the perfect candidate for Deep Learning applications and automation. However, spectroscopy is a high-dimensional and low sample domain, which introduces new issues for Deep Learning pipelines that are data hungry. To achieve state-of-the-art performance, deep models typically require large amounts of data that are difficult to collect, categorize, and manage in most domains. Data synthesis is a promising supplemental method to data acquisition that can reduce the cost and time overhead required to build such systems. This study sets out to identify the benefits and limitations of a statistical and deep generative approach to building synthetic data sets. The main focus was observing the general trends in learning when synthetic data were incorporated into the training phase of deep models.

For the task of identifying chlorinated solvents using blended data, CNN’s performance significantly improved by 23.5% up to 1200 samples but quickly converged with no further progress. The FCNN results are negligible, only obtaining a 1% increase in performance; however, the model robustness increased slightly reducing from 2.5% to 1% at 800 samples. The blended data had a positive effect for both methods, but it is evident that there is an upper limit on the number of samples that can be augmented before the performance diminishes. In the case of the SARS-CoV-2 task, the CNN exhibited a reverse effect, with the test evaluation scores decreasing after the augmentation at each step. We hypothesize that this decrease could be related to the insufficient representation of the data. Producing new spectra that share similar patterns and offer no new information is equivalent to extending the model training and allowing it to overfit naturally. Lastly, the SARS-CoV-2 FCNN experiment performed comparably to the blended model, obtaining a 1.2% increase in performance and a small increase in robustness.

Moving on to the VAE synthetic data, the chlorinated CNN model had a larger improvement of 26.1% at 2000 samples, and the FCNN model had an increase of only 3.5% at 1600 samples, which was only slightly higher than the improvement observed with the blended spectra. For both synthetic techniques, the chlorinated models received performance increases in all scenarios, likely due to the complexity and size of the data. In contrast, the SARS-CoV-2 models were negatively impacted, with 2.2–4% accuracy decreases for the CNN model and varying results for the FCNN, which achieved at most a 0.5% increase. What should also be considered is how the tasks were represented in the study. After augmentation, the chlorinated task results are comparable to those of the SARS-CoV-2 baseline due to the task being more complex and much larger. The results indicate that under the context of small, limited data sets neither task would achieve significant performance boosts by solely applying synthetic data.

We have demonstrated that synthetic methods have the ability to improve Deep Learning models and overcome issues such as overfitting and the curse of dimensionality. However, there is an upper limit on the performance gains and increased robustness of Deep Learning models when solely applying synthetic data. Other deep generative methods such as Generative Adversarial Networks (GANs) have been shown to outperform VAEs, but the design, training, and resources required are far greater than those of either method presented in this work. Synthetic data are not a valid substitute for high quality real-world data and should only be considered when a considerable amount of source data is available.

## Data Availability

This research was produced in Python v3.9.12 using an NVIDIA GeForce RTX 3080 and a 12th Gen Intel Core i9-12900K for training. The Deep Learning models are developed using open-source libraries TensorFlow v2.9.1 and Keras v2.9.0, and the weighted blending technique is implemented using NumPy v1.21.5 and Pandas v1.4.3. The python module similaritymeasures(39) is used for calculating the dFid. The source code and data required to run the algorithms are available on Github; refer to Springer Nature for the original SARS-CoV-2 spectra made publicly available by Yin et al.^11^

## Supporting Information Available

Experimental results of the two deep learning algorithms when augmented with synthetic spectra in increasing batch sizes. Includes the results of two data sets, one related to chlorinated data and the other involving the comparison of healthy individuals with positive SARS-CoV-2 patients (PDF)
