# Supplementary material for: A Comparative Analysis of Data Synthesis Techniques to Improve Classification Accuracy of Raman Spectroscopy Data
Source: J Chem Inf Model. 2023 Oct 11;64(7):2311–22. doi: 10.1021/acs.jcim.3c00761 (PMC11005048; doi:10.1021/acs.jcim.3c00761)
Supplement: Supplementary file 1 — ci3c00761_si_001.pdf [file ci3c00761_si_001.pdf]

Supporting Information

A Comparative Analysis of Data Synthesis Techniques to Improve Classification Accuracy of Raman Spectroscopy Data

Aaron R. Flanagan* and Frank G. Glavin*

School of Computer Science, University of Galway, Co. Galway, H91 FYH2, Ireland

A.flanagan18@universityofgalway.ie; frank.glavin@universityofgalway.ie

The following tables convey the results obtained from experiments conducted on two Deep Learning algorithms; a *Convolutional Neural Network (CNN) & Fully-Connected Neural Network (FCNN),* using two data sets; *chlorinated data* and *healthy vs positive SARS-CoV-2 patients.* Tables S1-S8 contain the test accuracy scores for the two data sets that are averaged over a 3-fold cross-validation. Each fold was trained on a new model and evaluated over five iterations. The Added samples column refers to the number of synthetic spectra augmented into the training fold.

Table S1. CNN test results for the chlorinated data augmented blended synthetic spectra

Added samples	Accuracy	Balanced Accuracy	Precision	Recall	F1-Score
0	58.8 ± 6.9	56.1 ± 4.1	72.2 ± 2.7	63.9 ± 14.6	66 ± 8.8
400	80.5 ± 2.8	77.4 ± 2.3	85 ± 1.4	86.5 ± 5.3	85.5 ± 2.5
800	82.2 ± 4.1	78.3 ± 5	85.3 ± 3.2	89.5 ± 3.9	87.1 ± 2.9
1200	82.5 ± 3.6	79.4 ± 4.5	86.2 ± 3.4	88.5 ± 3.7	87.1 ± 2.6
1600	82.6 ± 3.7	78.8 ± 4.5	85.8 ± 3.2	90 ± 3.8	87.4 ± 2.7
2000	82.1 ± 3.9	77.9 ± 5.5	84.7 ± 3.8	90.3 ± 2.5	87.2 ± 2.6

Table S2. FCNN test results for the chlorinated data augmented with blended synthetic spectra.

Added samples	Accuracy	Balanced Accuracy	Precision	Recall	F1-Score
0	77.8 ± 3	77.5 ± 2.5	87.8 ± 1.1	78.2 ± 4.5	82 ± 3.2
400	78.9 ± 1.2	77.7 ± 1.6	87.3 ± 1.4	81 ± 1.1	83.6 ± 0.9
800	79.5 ± 0.8	78.5 ± 1	87.8 ± 0.9	81.6 ± 0.6	84.2 ± 0.6
1200	79 ± 1	78.1 ± 1.1	87.9 ± 1	80.6 ± 1.4	83.6 ± 0.8
1600	78.7 ± 1.4	77.9 ± 1.1	87.9 ± 0.5	80.1 ± 2	83.2 ± 1.3
2000	78.7 ± 1.1	77.9 ± 1.1	87.9 ± 0.6	80.4 ± 1.7	83.4 ± 1

Table S3. CNN test results for the chlorinated data augmented with VAE synthetic spectra.

Added samples	Accuracy	Balanced Accuracy	Precision	Recall	F1-Score
0	58.8 ± 6.9	56.1 ± 4.1	72.2 ± 2.7	63.9 ± 14.6	66 ± 8.8
400	80.1 ± 4.1	76.9 ± 4.3	84.4 ± 2.9	86.4 ± 5.2	85.2 ± 3.3
800	82.1 ± 2.7	79.3 ± 3.4	86.1 ± 2.6	87.7 ± 4.2	86.7 ± 2.1
1200	83 ± 3.6	80 ± 3.9	86.5 ± 2.8	88.8 ± 4.3	87.4 ± 2.8
1600	83.2 ± 4.1	80.4 ± 5.5	87.5 ± 4.7	88.5 ± 4.9	87.6 ± 2.8
2000	84.7 ± 1.6	82.2 ± 1.8	87.9 ± 1.6	89.6 ± 2.5	88.7 ± 1.2

Table S4. FCNN test results for the chlorinated data augmented with VAE synthetic spectra.

Added samples	Accuracy	Balanced Accuracy	Precision	Recall	F1-Score
0	77.8 ± 3	77.5 ± 2.5	87.8 ± 1.1	78.2 ± 4.5	82 ± 3.2
400	81.6 ± 1.6	80.1 ± 1.7	88.2 ± 1.2	84.5 ± 1.7	86 ± 1.2
800	80.9 ± 1.7	79.8 ± 1.5	88.5 ± 0.7	82.9 ± 2.7	85.2 ± 1.6
1200	81.5 ± 2.3	80.3 ± 2.1	88.5 ± 1.3	83.8 ± 3.5	85.8 ± 2
1600	81.9 ± 1.7	80.7 ± 1.7	88.8 ± 1.5	84.2 ± 2.8	86.2 ± 1.5
2000	81.9 ± 2.3	81 ± 2.2	89.2 ± 1.2	83.9 ± 3.2	86.1 ± 2

Table S. CNN test results for the SARS-CoV-2 data augmented with blended synthetic spectra.

Added samples	Accuracy	Balanced Accuracy	Precision	Recall	F1-Score
0	86.2 ± 2	86.4 ± 2	93.7 ± 2.8	79.7 ± 1.9	85.7 ± 1.9
400	84.1 ± 2.2	84.2 ± 2.3	91.2 ± 2.5	80.4 ± 4.6	84 ± 2.1
800	85.1 ± 1.6	85.3 ± 1.6	92.2 ± 1.5	80.6 ± 3	84.8 ± 1.7
1200	85 ± 1.4	85.1 ± 1.4	90.6 ± 1.5	82.5 ± 2.9	85 ± 1.6
1600	85 ± 1.5	85.1 ± 1.5	90.8 ± 1.7	83.1 ± 1.5	85.3 ± 1.1
2000	84.9 ± 1.9	85 ± 1.9	91 ± 1.5	82.1 ± 3.2	84.9 ± 2

Table S6. FCNN test results for the SARS-CoV-2 data augmented with blended synthetic spectra.

Added samples	Accuracy	Balanced Accuracy	Precision	Recall	F1-Score
0	84.9 ± 2.8	84.9 ± 2.9	90.1 ± 2.4	84.2 ± 1.6	85.6 ± 2
400	85.9 ± 1	85.9 ± 1.1	91.3 ± 0.7	83.7 ± 1.4	86 ± 1.1
800	84.5 ± 3.6	84.5 ± 3.7	90.2 ± 2.9	83.3 ± 1.4	85.1 ± 2.6
1200	86 ± 1.1	86.1 ± 1.1	91.4 ± 0.7	83.8 ± 1.5	86.2 ± 1.2
1600	86 ± 1.2	86.1 ± 1.2	91.6 ± 0.9	83.6 ± 1.3	86.1 ± 1.2
2000	85.8 ± 1.3	85.9 ± 1.4	91.4 ± 1.3	83.5 ± 1	85.9 ± 1.2

Table S7. CNN test results for the SARS-CoV-2 data augmented with VAE synthetic spectra.

Added samples	Accuracy	Balanced Accuracy	Precision	Recall	F1-Score
0	86.2 ± 2	86.4 ± 2	93.7 ± 2.8	79.7 ± 1.9	85.7 ± 1.9
400	84.2 ± 2.6	84.2 ± 2.6	89.9 ± 2.9	82 ± 4.4	84.4 ± 2.6
800	83 ± 2	83.1 ± 2	89.4 ± 1.6	81.6 ± 3.3	83.5 ± 1.8
1200	82.8 ± 1.6	82.9 ± 1.5	89.7 ± 0.6	79.7 ± 3.6	82.8 ± 2
1600	82.9 ± 1.9	83 ± 1.9	89.4 ± 1.5	80.5 ± 3.7	83.1 ± 2
2000	82.3 ± 2.7	82.4 ± 2.7	88.8 ± 1.3	81 ± 3.2	82.9 ± 2.6

Table S8. FCNN test results for the SARS-CoV-2 data augmented with VAE synthetic spectra.

Added samples	Accuracy	Balanced Accuracy	Precision	Recall	F1-Score
0	84.9 ± 2.8	84.9 ± 2.9	90.1 ± 2.4	84.2 ± 1.6	85.6 ± 2
400	84.8 ± 1.6	84.9 ± 1.6	90.2 ± 1.2	83.5 ± 1.1	85.2 ± 1.4
800	85.1 ± 1.1	85.1 ± 1.1	90.6 ± 0.6	83.4 ± 1.6	85.5 ± 1.2
1200	84.8 ± 1	84.8 ± 1	89.9 ± 0.6	84.3 ± 1.5	85.5 ± 1
1600	85.4 ± 0.6	85.4 ± 0.6	90.8 ± 0.3	83.6 ± 1	85.7 ± 0.6
2000	84.6 ± 1.3	84.6 ± 1.3	90.1 ± 1.2	83.4 ± 0.5	85.1 ± 1.1
